# Mechanical Thrombectomy in Patients With Acute Ischemic Stroke: A Comparison of Transradial Versus Transfemoral Cerebral Angiography

**DOI:** 10.7759/cureus.10919

**Published:** 2020-10-12

**Authors:** Mohammad R Ghani, Vishal Busa, Ahmed Dardeir, Suganya Marudhai, Mauli Patel, Yousif M Abdelmoneim, Ahmad Jan, Noha Eskander

**Affiliations:** 1 Neurology, California Institute of Behavioral Neurosciences & Psychology, Fairfield, USA; 2 Internal Medicine/Family Medicine, California Institute of Behavioral Neurosciences & Psychology, Fairfield, USA; 3 Department of Physical Medicine and Rehabilitation, Richmond University Medical Center, New York, USA; 4 Internal Medicine, California Institute of Behavioral Neurosciences & Psychology, Fairfield, USA; 5 Department of Laboratory Medicine and Pathology, Mayo Clinic, Jacksonville, USA; 6 Surgery, California Institute of Behavioral Neurosciences & Psychology, Fairfield, USA; 7 Department of General Surgery and Emergency Services, International Medical Centre, Jeddah, SAU; 8 Psychiatry, California Institute of Behavioral Neurosciences & Psychology, Fairfield, USA

**Keywords:** acute ischemic stroke, cerebrovascular disease, mechanical thrombectomy, transradial access, transfemoral access

## Abstract

Stroke is the fourth leading cause of death in the United States and the primary reason for long-term disability. This debilitating condition can be divided into ischemic stroke and hemorrhagic stroke. The former occurs in almost 90% of all cases and arises from the occlusion of the supplying artery. Over the years, the management of stroke has developed from solely medical treatment to that which combines medical with mechanical treatment. Mechanical thrombectomy (MT) has drawn considerable interest in advanced medicine and is becoming more widely available. The two fundamental techniques in opening an occluded vessel are the transfemoral and transradial approaches. This literature review aims to compare the clinical implications, complication rate, and overall outcome between the transfemoral and transradial approaches in endovascular intervention in patients with acute ischemic stroke. We conducted a literature review on ischemic stroke and searched PubMed and Google Scholar for relevant articles published from January 2010 to March 2020. Mechanical thrombectomy has become the standard of care for patients with brain ischemia. The transradial approach exhibited superiority to the transfemoral route in resolving symptoms, decreased complication rates, and reduced healthcare costs in a subset of patients. In this literature review, the comparison between the two procedures reveals that the outcomes for anterior circulation stroke and posterior vascular system stroke may vary. Further research needs to be conducted to improve procedural skills and decrease technical difficulties, ultimately resulting in improved overall patient outcomes with respect to health and comfort.

## Introduction and background

With a prevalence of approximately 3% in the United States, stroke is the primary reason for long-term disability and the fourth leading cause of death [1]. Stroke can result from a ruptured vessel in the brain (hemorrhagic stroke) or blockage of blood supply (ischemic stroke) due to a thrombus or embolus [2]. Ischemic stroke is the most common type of stroke, comprising 90% of all cases. The blockage of blood flow limits the delivery of oxygen and nutrients, resulting in damage to or death of the brain cells, becoming permanent if the flow is not restored within a specified timeframe [2,3].

The circulation of the brain has two major arterial divisions, the anterior distribution, and posterior distribution. The anterior circulation supplies 80% of the brain circulation, arising from internal carotid arteries, whereas the posterior circulation supplies the remaining 20% of the brain circulation, specifically from vertebral arteries [4,5]. The anterior circulation branches into the middle cerebral artery (MCA) and anterior cerebral artery (ACA) [6]. The majority of acute ischemic stroke is due to large vessel occlusion in the anterior circulation, most frequently the internal carotid artery [6,7]. Management of ischemic stroke has advanced from conventional to interventional methods over the past decades, and modalities have been developed for different mechanical treatments. Among them, the commonly used methods are the transfemoral and transradial approaches for endovascular mechanical thrombectomy (MT), which is employed during acute ischemic stroke due to large vessel intracranial occlusion [8]. MT involves the delivery of a device into the affected blood vessel in the brain, typically through a catheter via the femoral artery, which then navigates toward the clot's location within the neurovasculature. This device is also used to capture and remove the clot [9]. MT has become the standard of care in the management of emergent large-vessel occlusive strokes.

The brachial, radial, transcervical, and direct carotid arteries are mostly used when access via the femoral artery is not possible. Neuroendovascular procedures are traditionally performed using the common transfemoral approach because of the size and length of endovascular equipment [10]. The transradial method has been extensively used as an alternative to the femoral approach in coronary interventions. Angiography via the transradial route was first described in 1989 by Campeau. This method was subsequently adopted, with its strengths and limitations identified [11]. In 2016, up to 4.5% of MT procedures in the US were performed via transradial access (TRA). Many studies reported on the benefits of this method, considering the comfort it provides for the patient, cost efficiency, and shortened hospital stay [12].

Over the years, stroke management has evolved from solely medical to combined medical and mechanical treatment. Before proceeding with an intervention, numerous factors have to be considered in determining the approach to implement. This narrative review aims to compare the clinical implications, complication rate, patient comfort, cost-effectiveness, and overall outcome of the transfemoral and transradial approaches to an endovascular intervention in adult patients with acute ischemic stroke [13].

## Review

Method

We conducted a literature search on PubMed and Google Scholar using the search words "mechanical thrombectomy", "acute ischemic stroke", "transradial angiography", "transfemoral angiography", "endovascular intervention". Reference lists of relevant articles identified using this method were scanned for other studies that were not identified through the electronic search. This resulted in more than 1680 articles in total. We reviewed 56 articles initially and 20 were included for the final reference list based on their relevance to the topics covered in this review. The studies published from January 2010 to March 2020 were included. The search was designed to identify studies regarding endovascular interventions via MT, particularly transradial and transfemoral thromboembolectomy, in patients with stroke. The search was limited to publications in English and studies conducted on humans. Exclusion criteria were interventions other than transradial or transfemoral, studies in another language other than English, non-humans studies, studies done outside the date assigned. The inclusion and exclusion criteria were set, and any disagreement was settled through a discussion. 

Results

Findings from some relevant studies that showed the role of the transradial and transfemoral approaches in the management of ischemic stroke are listed in Table 1.

**Table 1:** Summary of characteristics from some of the studies included. AIS: Acute ischemic stroke; PCI: percutaneous coronary intervention.

Author and publication year	Study design	Methodology	Diagnostic criteria	Conclusion
Balami JS, et al. (2018) [14]	Systematic review	Included only human studies and was limited to studies published in English between January 2014 and November 2016 based on relevance to the topics covered “Complications of endovascular treatment for acute ischemic stroke: Prevention and management” in the review.	Frequency of complications of mechanical thrombectomy in the treatment of acute ischemic stroke with an emphasis on perioperative complications.	The risk of complications with sequelae from mechanical thrombectomy was ∼15%, and the transfemoral approach was ineffective.
Jolly SS, et al. (2011) [15]	A randomized, parallel-group, multicenter trial	A total of 7021 patients were enrolled from 158 hospitals in 32 countries between June 6, 2006, and Nov 3, 2010; 3507 patients were randomly assigned to the radial access group and 3514 to the femoral access group.	Transradial and transfemoral approaches.	In this study with 3507 patients in the radial access arm vs 3514 in the femoral access arm. Transradial coronary angiography and angioplasty were safe, feasible, and effective with similar results to those of the transfemoral approach, (HR 0·49, 95% CI 0·28–0·87; p=0·015).
Haussen DC, et al. (2016) [16]	Retrospective review	A retrospective review of the local institutional AIS interventional databases of three tertiary academic centers.	Feasibility and safety of transradial access in the interventional management of acute ischemic stroke.	Failure of transfemoral access in the endovascular treatment of AIS was uncommon but led to unacceptable delays in reperfusion and poor outcomes. Standardization of benchmarks for access switches served as a guide for neuro interventionalists. Transfemoral access was a good approach for the endovascular treatment of acute ischemic stroke. Transradial access was effective in allowing clot engagement.
Mendiz OA, et al. (2016) [17]	Clinical trials	Clinical and angiographic data of 775 consecutive patients with high risk for carotid endarterectomy, treated between 1999 and 2016 by carotid artery stenting with cerebral protection.	Comparison of the outcome and complication rates of transradial and transfemoral carotid artery stenting.	Mechanical thrombectomy combined with standard intravenous thrombolysis improved functional independence in patients with acute cerebral ischemia, with no evidence of increased mortality. Bridging therapy should be considered for patients with large-vessel occlusions of the anterior circulation.
Bertrand OF, et al. (2010) [18]	Cross-sectional study	The survey was officially launched online on August 27, 2009, to collect 1,000 responses worldwide.	The survey was conducted from August 2009 to January 2010 among 1,107 interventional surgeons with extensive experience in transradial access in 75 countries.	Most respondents who used transradial access were moderate- or high-volume operators performing >100 PCIs/year.
Barros G, et al. (2020) [19]	Retrospective study	A retrospective chart review was prepared on patients who underwent cerebral angiography accessed via the left radial artery in three institutions from January 2018 to July 2019.	Technical feasibility of the left transradial access to cerebral angiography across three institutions.	Left transradial access in diagnostic and interventional cerebral angiography was a technically feasible, safe, and effective alternative when indicated. It would be preferable for situations in which pathologic locations or anatomic limitations preclude the right-sided radial access.
Zussman BM, et al. (2019) [20]	Clinical study	A subsequent prospective series of 50 consecutive right transradial diagnostic cerebral arteriograms were compared with initial institutional experience using a procedural staging system.	Prospective data on the learning curve for neuro interventionalists adopting this approach are limited.	Neurointerventionalists overcame the right transradial learning curve and achieved high success rates and low crossover rates after performing 30–50 cases.
Jo KW, et al. (2010) [21]	Clinical trials	From February 2007 to October 2009, 1,240 cerebral angiography procedures were performed via the single center's transradial approach.	Feasibility, efficacy, and safety of the transradial approach to cerebral angiography.	Cerebral angiography via the transradial approach with minimal risk of morbidity.
Chen SH, et al. (2019) [22]	A retrospective review of institutional database	A retrospective review of our institutional database to identify 51 patients with challenging vascular anatomy who underwent mechanical thrombectomy for anterior circulation large-vessel occlusion from February 2015 to February 2018.	Comparison of outcomes in patients who underwent mechanical thrombectomy via transradial access versus transfemoral access	Results demonstrate equivalence in efficacy and efficiency between transradial access and transfemoral access for the mechanical thrombectomy of the anterior circulation large-vessel occlusion in patients with challenging vascular anatomy.

Discussion

The common femoral artery remains the primary access site for many neuro interventionalists because of the large-caliber size, smooth compression to the femoral head, familiarity with the anatomy of the artery, and broad user experience with different catheters designs [23]. However, this traditional approach has several limitations. Anatomic factors such as aortoiliac occlusive disease and ectasia of the aorta, aortic arch, and supra-aortic vessels may hinder angiography and/or increase the risk of an intervention [24]. Other reasons that render the transfemoral approach less favorable than the transradial approach include the risk of complications related to the vascular system: retroperitoneal bleeding, arteriovenous fistulas, lower-extremity ischemia resulting from the dissection or baseline peripheral vascular disease, pseudoaneurysms, and femoral nerve damage [17,25]. Obese patients and those undergoing anticoagulation or antiplatelet therapy had a greater risk for complications with the transfemoral approach. Another disadvantage was that patients needed six hours of flatbed rest after the procedure in the femoral approach unless a percutaneous closure device was used [16].

Access through the transradial route had a vascular complication rate of 0.1%-0.2% [18,19]. Other complications that commonly occur with the transradial approach include asymptomatic temporary or permanent radial arterial occlusion, which has an occurrence rate of 5%. This occurrence is attributable to the small size of the radial artery prolonged cannulation, the radial artery diameter's ratio to the outer sheath diameter, and the anticoagulant used during arterial cannulation. Some reports suggest that these major complications stem from the length and large diameter of the introduced catheter. Therefore, avoiding a large and long catheter would prevent such major complications. Radial artery spasm can occur in approximately 10% of patients despite standardized preventative spasmolytic measures. This low rate is attributable to the collateral circulation of the hands [26]. Iatrogenic blockage of the radial artery is well tolerated in the presence of an intact palmar arcade and a competent ulnar artery. The patency of collaterals was evaluated before intervention by physical exam (Allen Test) and Doppler ultrasound [27]. In a study of 1360 patients, no radial artery occlusions occurred after immediate sheath removal. By contrast, 5% of the radial arteries became occluded when the sheath was left in place for more than three hours after the procedure [20].

Among the transradial cerebral intervention limitations is a high level of procedural skills required from the operator and the discomfort of performing such a procedure. The reasons the operator less prefers this approach include the training requirement, technical limitations associated with catheter technology, discomfort related to the laboratory staff or setup, apparently lengthy duration of the method, and procedural discomfort [21]. However, consistent learning and the experience of performing around 30-50 radial interventions can improve the skills of interventionalists, enhancing their efficiency and level of comfort with this procedure [28].

The radial artery location also facilitates hemostasis by local compression, minimizing risks for hematoma formation and damage to other structures [22]. Despite the minimal adverse effects associated with the transradial approach, conversion from the transradial to transfemoral access has been reported in 1%-7% of cases. A study reported higher failure rates in the transradial than the transfemoral and brachial routes for percutaneous transluminal coronary angioplasty, which was ascribed to radial artery spasm and narrow vessel caliber. Anatomical variations in the radial artery are not rare occurrences. In one study, almost 7.8% of 115 participants were found to have variable radial arteries. Variations such as a radioulnar loop, hypoplasia of the radial artery, or stenosis of the radial or brachial artery impede the successful completion of the endovascular procedure without necessarily disqualifying these patients from transradial procedures. The transradial method is also limited by the difficulty of accessing other arch vessels. Femoral access is considered if access to other arch vessels, particularly the contralateral vertebral artery, is needed [29].

About 71% of patients prefer TRA over the transfemoral route when presented with the option. This preference is attributable to the higher bleeding risk, a higher degree of pain during the procedure, and within hours after the procedure, and lower tolerance for postoperative mobility precautions associated with the transfemoral approach. After a transradial procedure, patients can walk immediately, increasing patient comfort. Decreases in hospitalization time and adverse outcomes associated with TRA also translated into reduced healthcare costs [25].

Another significant advantage of TRA is that it allows the performance of angiography after anticoagulation therapy with increased hemostatic control. Patients with coagulopathies or receiving anticoagulation therapy were suitable candidates for TRA because of their higher risk for bleeding complications with transfemoral approach (TFA), precisely when no closure device was used. 

The advantages and disadvantages of TFA and TRA in acute ischemic stroke are summarized in Table 2 [18,19,25]. 

**Table 2:** Summary of the advantages and disadvantages of transfemoral access and transradial access in acute ischemic stroke.

Transradial Approach		Transfemoral Approach	
Advantages	Disadvantages	Advantages	Disadvantages
- Lower morbidity and mortality compared with the transfemoral approach - Low risk of vascular complications - it is the preferable method for a patient on anticoagulant or antiplatelet therapy - Low procedural cost - Improved recovery time and early discharge - Less pain with arterial puncture intraoperatively and postoperatively - Presence of collaterals can compensate for radial arterial occlusion - Discontinuation of the nothing-by-mouth restriction shortly after the procedure - Greater patient satisfaction than the transfemoral approach	- Limited operator experience - Difficulty of the procedure due to anatomical variations - Risk of iatrogenic stenosis - Varying levels of difficulty depending on the diameter of the radial artery - Longer duration compared with the femoral approach - Technical limitation attributable to catheter technology and discomfort related to laboratory staff/setup	- Availability of interventionalist with experience in this procedure - Large femoral artery diameter - Known and manageable procedural complications - Preferable method in patients with peripheral arterial disease	- Vascular complications, including bleeding, pseudoaneurysms, and clot formation - Extended hospital stays - Lower patient satisfaction and higher patient discomfort compared with the transradial approach - Higher procedural costs involved compared with the transradial approach - Femoral artery is the only source of blood to the leg

## Conclusions

In the last decade, the transradial and transfemoral approaches in ischemic stroke management have been largely studied and drawn significant interest. Although both techniques exhibit efficiency, practicality, and benefits, they also involved complications. In this narrative literature review, we demonstrate the expediency of TRA over TFA for endovascular interventions in patients with ischemic stroke. TRA presents several advantages but also has limitations. Overall, the use of TRA has gained preference by doctors and patients, but TFA remains as the first option in most cases.

Limitations and future directions

One of the challenges encountered in this study is that the procedures described are not categorized for anterior or posterior circulation stroke. The duration of the complications encountered during either technique was not measured, and the long-term benefits or outcomes were not identified. Moreover, the catheter technique used was not always clear. Accordingly, we recommend conducting further studies to improve the catheter technique and operator procedural skills, leading to improved outcomes in patient health and comfort. 
